# Supplementary material for: A new giant sauropod, Australotitan cooperensis gen. et sp. nov., from the mid-Cretaceous of Australia
Source: PeerJ. 2021 Jun 7;9:e11317. doi: 10.7717/peerj.11317 (PMC8191491; doi:10.7717/peerj.11317)
Supplement: Supplemental Information 4 [file peerj-09-11317-s004.pdf]

Supplementary Information

Hocknull et al. 2021. A new giant sauropod, *Australotitan cooperensis* gen. et sp. nov., from the mid-Cretaceous of Australia.

Character with character state changes for Wintonotitan wattsi, Diamantinasaurus matildae and Savannasaurus elliottorum.

Characters from Royo-Torres et al, 2021 & Poropat et al 2021	Taxon	Former State	New State	Estimated State	Justification
36	*A. cooperensis*<br>*W. wattsi*			0<br>0	Estimated based on reconstructed scapulae.
37	*Au. cooperensis*<br>*W. wattsi*<br>*D. matildae*			0<br>0<br>0	Estimated based on reconstructed scapulae.
40	*D. matildae*			1	Estimate based on reconstructed humerus.
41	*Au. cooperensis*<br>*D. matildae*<br>*W. wattsi*			0<br>0<br>0/1	Estimate based on reconstructed humerus<br>Estimate based on reconstructed humerus<br>Estimate is close to 0.4, therefore could be either 0 or 1
43	*D. matildae*	1	0		Humeral shaft eccentricity <3.0 (2.8)
45	*W. wattsi*	0	?		Not preserved, estimate difficult
46	*W. wattsi*	1	?		Not preserved, estimate difficult
50	*W. wattsi*	0	?	0	Not well preserved, estimated
51	*D. matildae*	1	0		Ratio = 1.11, <1/4 = 0
52	*W. wattsi*	1	?		Not preserved, estimate difficult

53	*W. wattsi*	0	?		Not preserved, estimate difficult
58	*D. matildae*<br>*S. elliottorum*	0<br>1	1<br>?		Ratio 2.63/2.81 = 1<br>Not preserved, estimate difficult
59	*D. matildae*	1	0/1		0.39 ratio close to range definition so include both possibilities
62	*D. matildae*<br>*S. elliottorum*<br>*W. wattsi*	0<br>0<br>0	1<br>1<br>?		0.85 = 1<br>0.83 = 1<br>Not preserved, estimate difficult
217	*W. wattsi*	0	1		D-shaped scapular base cross-section, equivalent to *D. matildae*
224	*S. elliottorum*	1	0		Concave lateral margin of humeral diaphysis present.
228	*S. elliottorum*<br>*W. wattsi*	1<br>0	?<br>?	1<br>1	Not preserved, estimated to have a deeply concave (1) as in *D. matildae*.
229	*W. wattsi*	0	?		Not preserved, estimate difficult
230	*W. wattsi*	0	?		Not preserved, estimate difficult
233	*W. wattsi*	0	?	1	Not well preserved, estimated to project above proximal articulation (1).
236	*W. wattsi*	1	?	0	Not preserved, estimated to be prominently expanded posteriorly similar to *D. matildae*.
252	*W. wattsi*	0	?	0	Not well preserved, predicted to be strongly concave similar to *D. matildae* and *A. cooperensis*.
282	*W. wattsi*	0	?		Not preserved, estimate difficult
365	*W. wattsi*<br>*S. elliottorum*	?<br>?	?<br>?	1<br>1	Not preserved, estimated overall shape.<br>Not preserved, estimated overall shape.
367	*D. matildae*	1	?	?	Cannot define a vertical ridge.

369	*W. wattsi*	0	?		Not preserved, estimate difficult
386	*W. wattsi*<br>*D. matildae*	0<br>1	?<br>0		Not preserved, estimate difficult<br>Ratio = 1.96 >1.0 = 0
413	*W. wattsi*	0	?		Not preserved, estimate difficult
511	*W. wattsi*	0	?		Not preserved, estimate difficult
513	*W. wattsi*<br>*D. matildae*	0<br>?	1<br>1		Muscle scar / tuberosity present<br>Muscle scar / tuberosity present
514	*D. matildae*<br>*W. wattsi*	?<br>?	?<br>?	1<br>1	<5.5 (1)<br><5.5 (1)
515	*W. wattsi*<br>*A. cooperensis*	?<br>?	?<br>?	0<br>0	Not preserved, estimated<br>Not preserved, estimated
516	*W. wattsi*	?	?	0	Not preserved, estimated from reconstructed humerus
521	*W. wattsi*<br>*D. matildae*<br>*S. elliottorum*	0<br>0<br>0	?<br>1<br>?		Not preserved, estimate difficult<br>Meets at right angle<br>Not preserved, estimate difficult
533	*W. wattsi*	?	?	1	Not preserved, estimated based on ischial similarities with *D. matildae*, *A. cooperensis*.
