# Supplementary material for: A new giant sauropod, Australotitan cooperensis gen. et sp. nov., from the mid-Cretaceous of Australia
Source: PeerJ. 2021 Jun 7;9:e11317. doi: 10.7717/peerj.11317 (PMC8191491; doi:10.7717/peerj.11317)
Supplement: Supplemental Information 5 — Results from preliminary assessment of phylogenetic position of Australotitan cooperensis gen. et sp. nov. within the datasets of Poropat et al. (2021) and Royo-Torres et al. (2021). [file peerj-09-11317-s005.pdf]

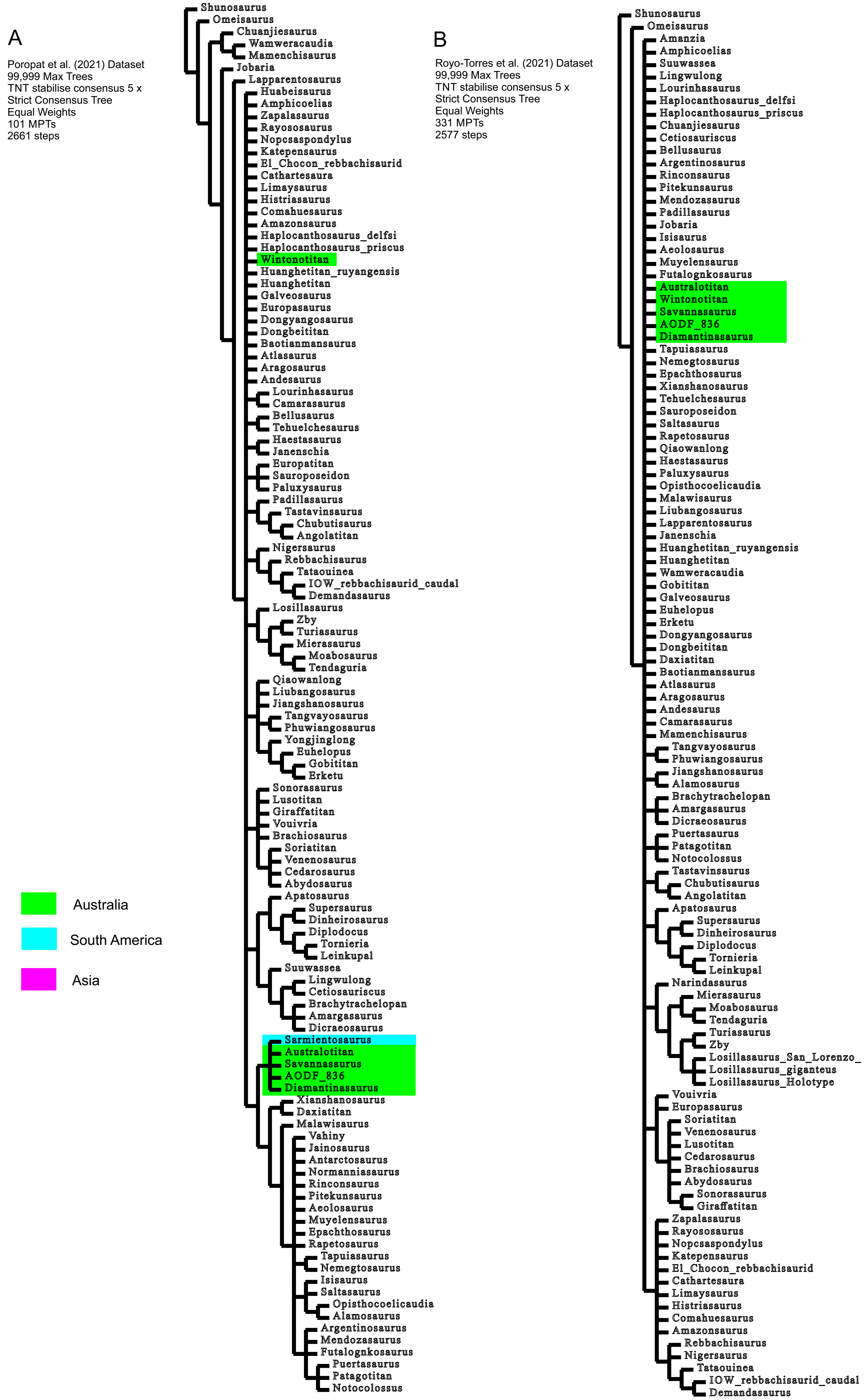

A
Poropat et al. (2021) Dataset
99,999 Max Trees
TNT stabilise consensus 5 x
Strict Consensus Tree
Equal Weights
101 MPTs
2661 steps
Shunosaurus
Omeisaurus
Chuanjiesaurus
Wamweracaudia
Mamenchisaurus
Jobaria
Lapparentosaurus
Huabeisaurus
Amphicoelias
Zapalasaurus
Rayososaurus
Nopcsaspondylus
Katepensaurus
El_Chocon_rebbachisaurid
Cathartesaura
Limaysaurus
Histriasaurus
Comahuesaurus
Amazonsaurus
Haplocanthosaurus_delfsi
Haplocanthosaurus_priscus
Wintonotitan
Huanghetitan_ruyangensis
Huanghetitan
Galveosaurus
Europasaurus
Dongyangosaurus
Dongbeititan
Baotianmansaurus
Atlasaurus
Aragosaurus
Andesaurus
Lourinhasaurus
Camarasaurus
Bellusaurus
Tehuelchesaurus
Haestasaurus
Janenschia
Europatitan
Sauroposeidon
Paluxysaurus
Padillasaurus
Tastavinsaurus
Chubutisaurus
Angolatitan
Nigersaurus
Rebbachisaurus
Tataouinea
IOW_rebbachisaurid_caudal
Demandasaurus
Losillasaurus
Zby
Turiasaurus
Mierasaurus
Moabosaurus
Tendaguria
Qiaowanlong
Liubangosaurus
Jiangshanosaurus
Tangvayosaurus
Phuwiangosaurus
Yongjinglong
Euhelopus
Gobititan
Erketu
Sonorasaurus
Lusotitan
Giraffatitan
Vouivria
Brachiosaurus
Soriatitan
Venenosaurus
Cedarosaurus
Abydosaurus
Apatosaurus
Supersaurus
Dinheirosaurus
Diplodocus
Tornieria
Leinkupal
Suuwassea
Lingwulong
Cetiosauriscus
Brachytrachelopan
Amargasaurus
Dicraeosaurus
Sarmientosaurus
Australotitan
Savannasaurus
AODF_836
Diamantinasaurus
Xianshanosaurus
Daxiatitan
Malawisaurus
Vahiny
Jainosaurus
Antarctosaurus
Normanniasaurus
Rinconsaurus
Pitekunsaurus
Aeolosaurus
Muyelensaurus
Epachthosaurus
Rapetosaurus
Tapuiasaurus
Nemegtosaurus
Isisaurus
Saltasaurus
Opisthocoelicaudia
Alamosaurus
Argentinosaurus
Mendozasaurus
Futalognkosaurus
Puertasaurus
Patagotitan
Notocolossus
Australia
South America
Asia
B
Royo-Torres et al. (2021) Dataset
99,999 Max Trees
TNT stabilise consensus 5 x
Strict Consensus Tree
Equal Weights
331 MPTs
2577 steps
Shunosaurus
Omeisaurus
Amanzia
Amphicoelias
Suuwassea
Lingwulong
Lourinhasaurus
Haplocanthosaurus_delfsi
Haplocanthosaurus_priscus
Chuanjiesaurus
Cetiosauriscus
Bellusaurus
Argentinosaurus
Rinconsaurus
Pitekunsaurus
Mendozasaurus
Padillasaurus
Jobaria
Isisaurus
Aeolosaurus
Muyelensaurus
Futalognkosaurus
Australotitan
Wintonotitan
Savannasaurus
AODF_836
Diamantinasaurus
Tapuiasaurus
Nemegtosaurus
Epachthosaurus
Xianshanosaurus
Tehuelchesaurus
Sauroposeidon
Saltasaurus
Rapetosaurus
Qiaowanlong
Haestasaurus
Paluxysaurus
Opisthocoelicaudia
Malawisaurus
Liubangosaurus
Lapparentosaurus
Janenschia
Huanghetitan_ruyangensis
Huanghetitan
Wamweracaudia
Gobititan
Galveosaurus
Euhelopus
Erketu
Dongyangosaurus
Dongbeititan
Daxiatitan
Baotianmansaurus
Atlasaurus
Aragosaurus
Andesaurus
Camarasaurus
Mamenchisaurus
Tangvayosaurus
Phuwiangosaurus
Jiangshanosaurus
Alamosaurus
Brachytrachelopan
Amargasaurus
Dicraeosaurus
Puertasaurus
Patagotitan
Notocolossus
Tastavinsaurus
Chubutisaurus
Angolatitan
Apatosaurus
Supersaurus
Dinheirosaurus
Diplodocus
Tornieria
Leinkupal
Narindasaurus
Mierasaurus
Moabosaurus
Tendaguria
Turiasaurus
Zby
Losillasaurus_San_Lorenzo_
Losillasaurus_giganteus
Losillasaurus_Holotype
Vouivria
Europasaurus
Soriatitan
Venenosaurus
Lusotitan
Cedarosaurus
Brachiosaurus
Abydosaurus
Sonorasaurus
Giraffatitan
Zapalasaurus
Rayososaurus
Nopcsaspondylus
Katepensaurus
El_Chocon_rebbachisaurid
Cathartesaura
Limaysaurus
Histriasaurus
Comahuesaurus
Amazonsaurus
Rebbachisaurus
Nigersaurus
Tataouinea
IOW_rebbachisaurid_caudal
Demandasaurus

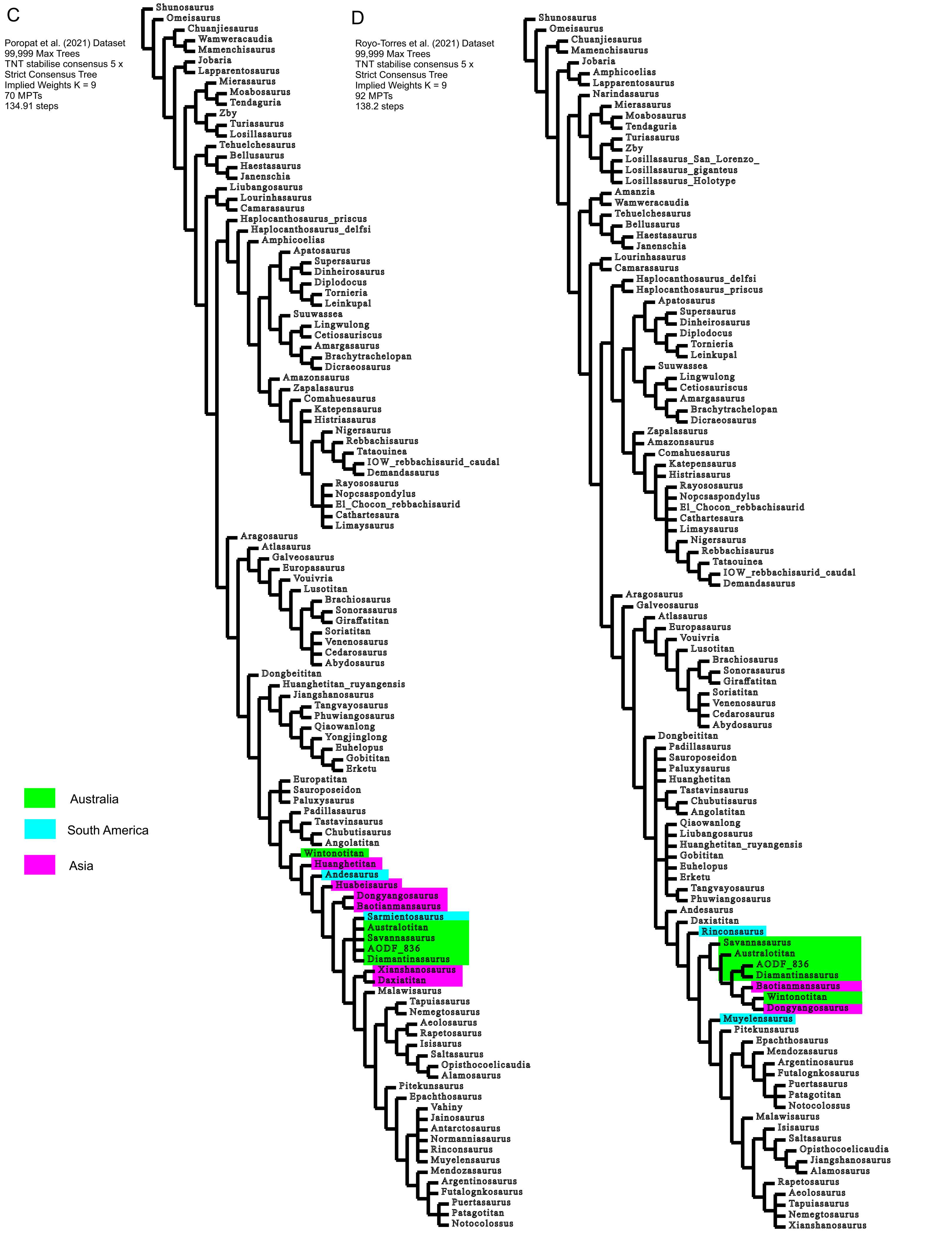

C
Poropat et al. (2021) Dataset
99,999 Max Trees
TNT stabilise consensus 5 x
Strict Consensus Tree
Implied Weights K = 9
70 MPTs
134.91 steps
Shunosaurus
Omeisaurus
Chuanjiesaurus
Wamweracaudia
Mamenchisaurus
Jobaria
Lapparentosaurus
Mierasaurus
Moabosaurus
Tendaguria
Zby
Turiasaurus
Losillasaurus
Tehuelchesaurus
Bellusaurus
Haestasaurus
Janenschia
Liubangosaurus
Lourinhasaurus
Camarasaurus
Haplocanthosaurus_priscus
Haplocanthosaurus_delfsi
Amphicoelias
Apatosaurus
Supersaurus
Dinheirosaurus
Diplodocus
Tornieria
Leinkupal
Suuwassea
Lingwulong
Cetiosauriscus
Amargasaurus
Brachytrachelopan
Dicraeosaurus
Amazonsaurus
Zapalasaurus
Comahuesaurus
Katepensaurus
Histriasaurus
Nigersaurus
Rebbachisaurus
Tataouinea
IOW_rebbachisaurid_caudal
Demandasaurus
Rayososaurus
Nopcsaspondylus
El_Chocon_rebbachisaurid
Cathartesaura
Limaysaurus
Aragosaurus
Atlasaurus
Galveosaurus
Europasaurus
Vouivria
Lusotitan
Brachiosaurus
Sonorasaurus
Giraffatitan
Soriatitan
Venenosaurus
Cedarosaurus
Abydosaurus
Dongbeititan
Huanghetitan_ruyangensis
Jiangshanosaurus
Tangvayosaurus
Phuwiangosaurus
Qiaowanlong
Yongjinglong
Euhelopus
Gobititan
Erketu
Europatitan
Sauroposeidon
Paluxysaurus
Padillasaurus
Tastavinsaurus
Chubutisaurus
Angolatitan
Wintonotitan
Huanghetitan
Andesaurus
Huabeisaurus
Dongyangosaurus
Baotianmansaurus
Sarmientosaurus
Australotitan
Savannasaurus
AODF_836
Diamantinasaurus
Xianshanosaurus
Daxiatitan
Malawisaurus
Tapuiasaurus
Nemegtosaurus
Aeolosaurus
Rapetosaurus
Isisaurus
Saltasaurus
Opisthocoelicaudia
Alamosaurus
Pitekunsaurus
Epachthosaurus
Vahiny
Jainosaurus
Antarctosaurus
Normanniasaurus
Rinconsaurus
Muyelensaurus
Mendozasaurus
Argentinosaurus
Futalognkosaurus
Puertasaurus
Patagotitan
Notocolossus
Australia
South America
Asia
D
Royo-Torres et al. (2021) Dataset
99,999 Max Trees
TNT stabilise consensus 5 x
Strict Consensus Tree
Implied Weights K = 9
92 MPTs
138.2 steps
Shunosaurus
Omeisaurus
Chuanjiesaurus
Mamenchisaurus
Jobaria
Amphicoelias
Lapparentosaurus
Narindasaurus
Mierasaurus
Moabosaurus
Tendaguria
Turiasaurus
Zby
Losillasaurus_San_Lorenzo_
Losillasaurus_giganteus
Losillasaurus_Holotype
Amanzia
Wamweracaudia
Tehuelchesaurus
Bellusaurus
Haestasaurus
Janenschia
Lourinhasaurus
Camarasaurus
Haplocanthosaurus_delfsi
Haplocanthosaurus_priscus
Apatosaurus
Supersaurus
Dinheirosaurus
Diplodocus
Tornieria
Leinkupal
Suuwassea
Lingwulong
Cetiosauriscus
Amargasaurus
Brachytrachelopan
Dicraeosaurus
Zapalasaurus
Amazonsaurus
Comahuesaurus
Katepensaurus
Histriasaurus
Rayososaurus
Nopcsaspondylus
El_Chocon_rebbachisaurid
Cathartesaura
Limaysaurus
Nigersaurus
Rebbachisaurus
Tataouinea
IOW_rebbachisaurid_caudal
Demandasaurus
Aragosaurus
Galveosaurus
Atlasaurus
Europasaurus
Vouivria
Lusotitan
Brachiosaurus
Sonorasaurus
Giraffatitan
Soriatitan
Venenosaurus
Cedarosaurus
Abydosaurus
Dongbeititan
Padillasaurus
Sauroposeidon
Paluxysaurus
Huanghetitan
Tastavinsaurus
Chubutisaurus
Angolatitan
Qiaowanlong
Liubangosaurus
Huanghetitan_ruyangensis
Gobititan
Euhelopus
Erketu
Tangvayosaurus
Phuwiangosaurus
Andesaurus
Daxiatitan
Rinconsaurus
Savannasaurus
Australotitan
AODF_836
Diamantinasaurus
Baotianmansaurus
Wintonotitan
Dongyangosaurus
Muyelensaurus
Pitekunsaurus
Epachthosaurus
Mendozasaurus
Argentinosaurus
Futalognkosaurus
Puertasaurus
Patagotitan
Notocolossus
Malawisaurus
Isisaurus
Saltasaurus
Opisthocoelicaudia
Jiangshanosaurus
Alamosaurus
Rapetosaurus
Aeolosaurus
Tapuiasaurus
Nemegtosaurus
Xianshanosaurus

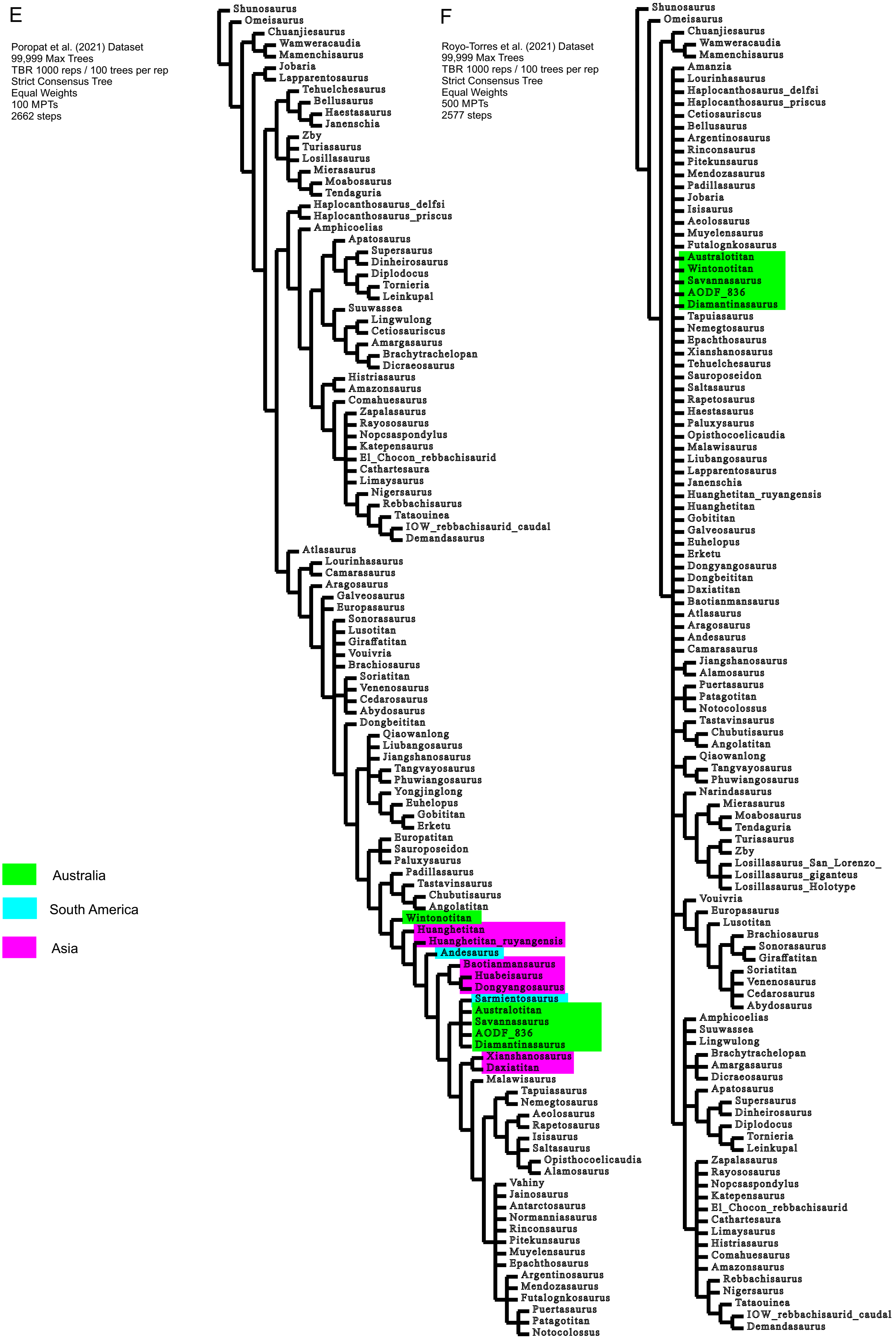
E
Poropat et al. (2021) Dataset
99,999 Max Trees
TBR 1000 reps / 100 trees per rep
Strict Consensus Tree
Equal Weights
100 MPTs
2662 steps
Shunosaurus
Omeisaurus
Chuanjiesaurus
Wamweracaudia
Mamenchisaurus
Jobaria
Lapparentosaurus
Tehuelchesaurus
Bellusaurus
Haestasaurus
Janenschia
Zby
Turiasaurus
Losillasaurus
Mierasaurus
Moabosaurus
Tendaguria
Haplocanthosaurus_delfsi
Haplocanthosaurus_priscus
Amphicoelias
Apatosaurus
Supersaurus
Dinheirosaurus
Diplodocus
Tornieria
Leinkupal
Suuwassea
Lingwulong
Cetiosauriscus
Amargasaurus
Brachytrachelopan
Dicraeosaurus
Histriasaurus
Amazonsaurus
Comahuesaurus
Zapalasaurus
Rayososaurus
Nopcsaspondylus
Katepensaurus
El_Chocon_rebbachisaurid
Cathartesaura
Limaysaurus
Nigersaurus
Rebbachisaurus
Tataouinea
IOW_rebbachisaurid_caudal
Demandasaurus
Atlasaurus
Lourinhasaurus
Camarasaurus
Aragosaurus
Galveosaurus
Europasaurus
Sonorasaurus
Lusotitan
Giraffatitan
Vouivria
Brachiosaurus
Soriatitan
Venenosaurus
Cedarosaurus
Abydosaurus
Dongbeititan
Qiaowanlong
Liubangosaurus
Jiangshanosaurus
Tangvayosaurus
Phuwiangosaurus
Yongjinglong
Euhelopus
Gobititan
Erketu
Europatitan
Sauroposeidon
Paluxysaurus
Padillasaurus
Tastavinsaurus
Chubutisaurus
Angolatitan
Wintonotitan
Huanghetitan
Huanghetitan_ruyangensis
Andesaurus
Baotianmansaurus
Huabeisaurus
Dongyangosaurus
Sarmientosaurus
Australotitan
Savannasaurus
AODF_836
Diamantinasaurus
Xianshanosaurus
Daxiatitan
Malawisaurus
Tapuiasaurus
Nemegtosaurus
Aeolosaurus
Rapetosaurus
Isisaurus
Saltasaurus
Opisthocoelicaudia
Alamosaurus
Vahiny
Jainosaurus
Antarctosaurus
Normanniasaurus
Rinconsaurus
Pitekunsaurus
Muyelensaurus
Epachthosaurus
Argentinosaurus
Mendozasaurus
Futalognkosaurus
Puertasaurus
Patagotitan
Notocolossus
F
Royo-Torres et al. (2021) Dataset
99,999 Max Trees
TBR 1000 reps / 100 trees per rep
Strict Consensus Tree
Equal Weights
500 MPTs
2577 steps
Shunosaurus
Omeisaurus
Chuanjiesaurus
Wamweracaudia
Mamenchisaurus
Amanzia
Lourinhasaurus
Haplocanthosaurus_delfsi
Haplocanthosaurus_priscus
Cetiosauriscus
Bellusaurus
Argentinosaurus
Rinconsaurus
Pitekunsaurus
Mendozasaurus
Padillasaurus
Jobaria
Isisaurus
Aeolosaurus
Muyelensaurus
Futalognkosaurus
Australotitan
Wintonotitan
Savannasaurus
AODF_836
Diamantinasaurus
Tapuiasaurus
Nemegtosaurus
Epachthosaurus
Xianshanosaurus
Tehuelchesaurus
Sauroposeidon
Saltasaurus
Rapetosaurus
Haestasaurus
Paluxysaurus
Opisthocoelicaudia
Malawisaurus
Liubangosaurus
Lapparentosaurus
Janenschia
Huanghetitan_ruyangensis
Huanghetitan
Gobititan
Galveosaurus
Euhelopus
Erketu
Dongyangosaurus
Dongbeititan
Daxiatitan
Baotianmansaurus
Atlasaurus
Aragosaurus
Andesaurus
Camarasaurus
Jiangshanosaurus
Alamosaurus
Puertasaurus
Patagotitan
Notocolossus
Tastavinsaurus
Chubutisaurus
Angolatitan
Qiaowanlong
Tangvayosaurus
Phuwiangosaurus
Narindasaurus
Mierasaurus
Moabosaurus
Tendaguria
Turiasaurus
Zby
Losillasaurus_San_Lorenzo_
Losillasaurus_giganteus
Losillasaurus_Holotype
Vouivria
Europasaurus
Lusotitan
Brachiosaurus
Sonorasaurus
Giraffatitan
Soriatitan
Venenosaurus
Cedarosaurus
Abydosaurus
Amphicoelias
Suuwassea
Lingwulong
Brachytrachelopan
Amargasaurus
Dicraeosaurus
Apatosaurus
Supersaurus
Dinheirosaurus
Diplodocus
Tornieria
Leinkupal
Zapalasaurus
Rayososaurus
Nopcsaspondylus
Katepensaurus
El_Chocon_rebbachisaurid
Cathartesaura
Limaysaurus
Histriasaurus
Comahuesaurus
Amazonsaurus
Rebbachisaurus
Nigersaurus
Tataouinea
IOW_rebbachisaurid_caudal
Demandasaurus
Australia
South America
Asia

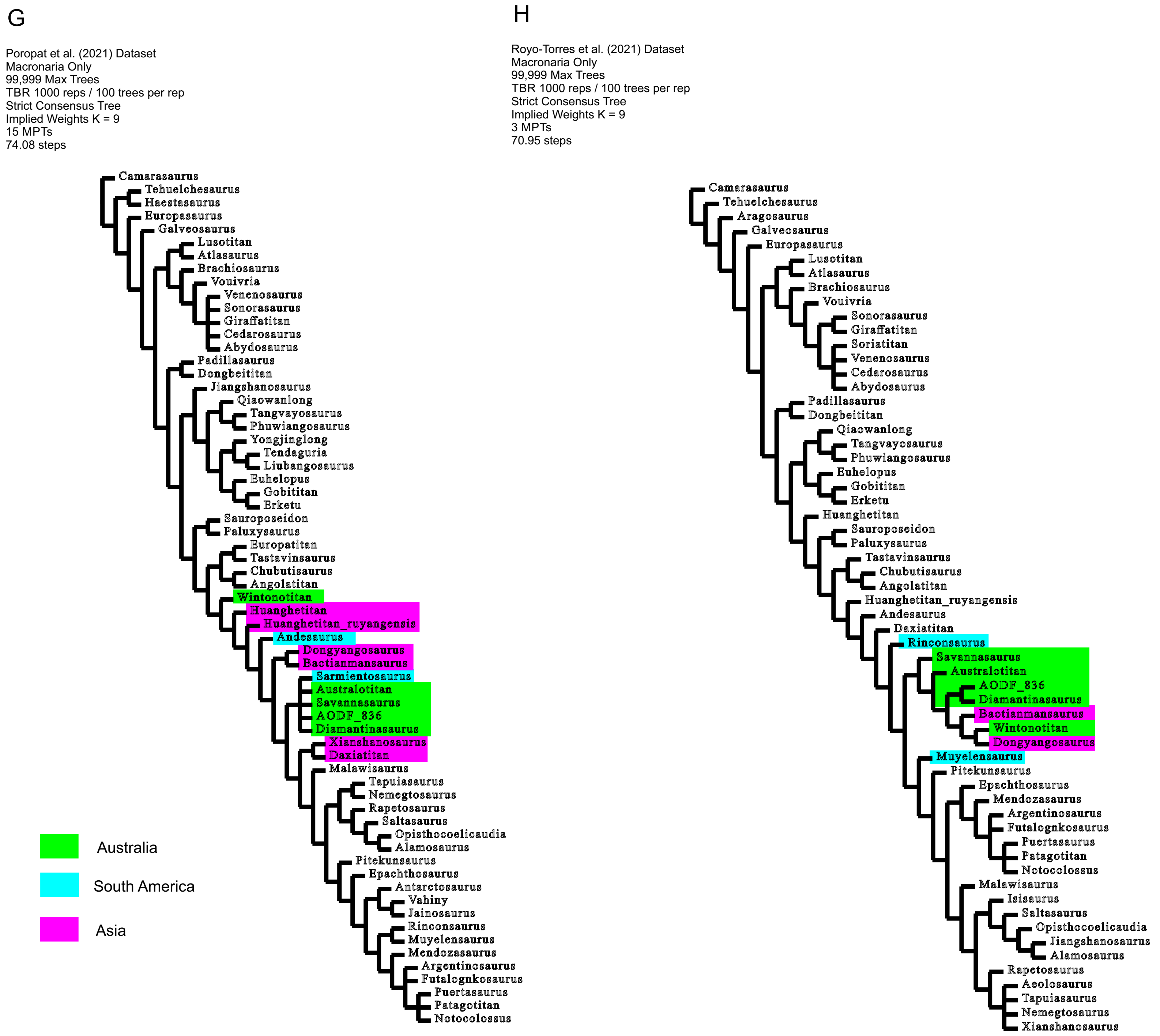
G
Poropat et al. (2021) Dataset
Macronaria Only
99,999 Max Trees
TBR 1000 reps / 100 trees per rep
Strict Consensus Tree
Implied Weights K = 9
15 MPTs
74.08 steps
Camarasaurus
Tehuelchesaurus
Haestasaurus
Europasaurus
Galveosaurus
Lusotitan
Atlasaurus
Brachiosaurus
Vouivria
Venenosaurus
Sonorasaurus
Giraffatitan
Cedarosaurus
Abydosaurus
Padillasaurus
Dongbeititan
Jiangshanosaurus
Qiaowanlong
Tangvayosaurus
Phuwiangosaurus
Yongjinglong
Tendaguria
Liubangosaurus
Euhelopus
Gobititan
Erketu
Sauroposeidon
Paluxysaurus
Europatitan
Tastavinsaurus
Chubutisaurus
Angolatitan
Wintonotitan
Huanghetitan
Huanghetitan_ruyangensis
Andesaurus
Dongyangosaurus
Baotianmansaurus
Sarmientosaurus
Australotitan
Savannasaurus
AODF_836
Diamantinasaurus
Xianshanosaurus
Daxiatitan
Malawisaurus
Tapuiasaurus
Nemegtosaurus
Rapetosaurus
Saltasaurus
Opisthocoelicaudia
Alamosaurus
Pitekunsaurus
Epachthosaurus
Antarctosaurus
Vahiny
Jainosaurus
Rinconsaurus
Muyelensaurus
Mendozasaurus
Argentinosaurus
Futalognkosaurus
Puertasaurus
Patagotitan
Notocolossus
Australia
South America
Asia
H
Royo-Torres et al. (2021) Dataset
Macronaria Only
99,999 Max Trees
TBR 1000 reps / 100 trees per rep
Strict Consensus Tree
Implied Weights K = 9
3 MPTs
70.95 steps
Camarasaurus
Tehuelchesaurus
Aragosaurus
Galveosaurus
Europasaurus
Lusotitan
Atlasaurus
Brachiosaurus
Vouivria
Sonorasaurus
Giraffatitan
Soriatitan
Venenosaurus
Cedarosaurus
Abydosaurus
Padillasaurus
Dongbeititan
Qiaowanlong
Tangvayosaurus
Phuwiangosaurus
Euhelopus
Gobititan
Erketu
Huanghetitan
Sauroposeidon
Paluxysaurus
Tastavinsaurus
Chubutisaurus
Angolatitan
Huanghetitan_ruyangensis
Andesaurus
Daxiatitan
Rinconsaurus
Savannasaurus
Australotitan
AODF_836
Diamantinasaurus
Baotianmansaurus
Wintonotitan
Dongyangosaurus
Muyelensaurus
Pitekunsaurus
Epachthosaurus
Mendozasaurus
Argentinosaurus
Futalognkosaurus
Puertasaurus
Patagotitan
Notocolossus
Malawisaurus
Isisaurus
Saltasaurus
Opisthocoelicaudia
Jiangshanosaurus
Alamosaurus
Rapetosaurus
Aeolosaurus
Tapuiasaurus
Nemegtosaurus
Xianshanosaurus

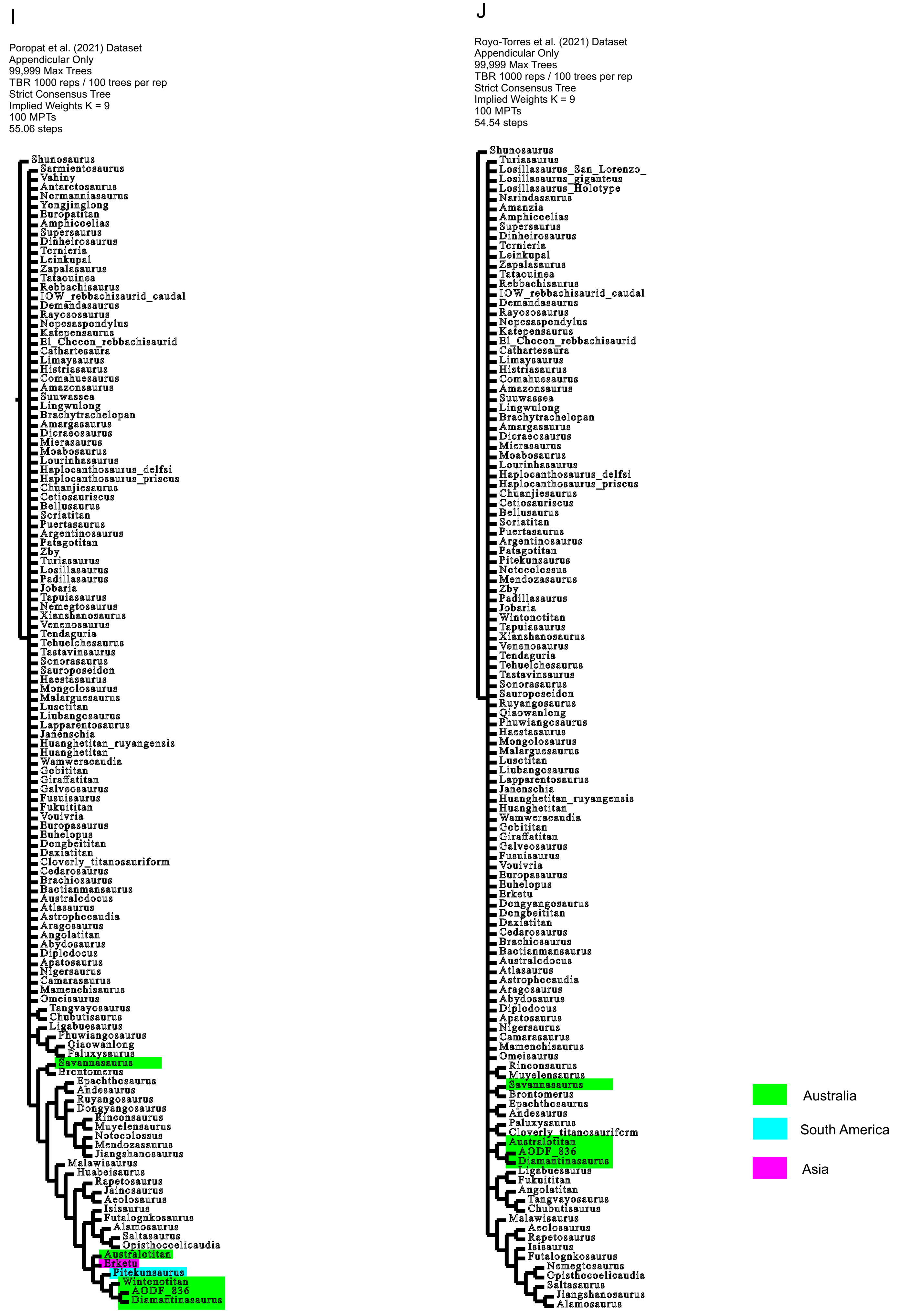

I
Poropat et al. (2021) Dataset
Appendicular Only
99,999 Max Trees
TBR 1000 reps / 100 trees per rep
Strict Consensus Tree
Implied Weights K = 9
100 MPTs
55.06 steps
Shunosaurus
Sarmientosaurus
Vahiny
Antarctosaurus
Normanniasaurus
Yongjinglong
Europatitan
Amphicoelias
Supersaurus
Dinheirosaurus
Tornieria
Leinkupal
Zapalasaurus
Tataouinea
Rebbachisaurus
IOW_rebbachisaurid_caudal
Demandasaurus
Rayososaurus
Nopcsaspondylus
Katepensaurus
El_Chocon_rebbachisaurid
Cathartesaura
Limaysaurus
Histriasaurus
Comahuesaurus
Amazonsaurus
Suuwassea
Lingwulong
Brachytrachelopan
Amargasaurus
Dicraeosaurus
Mierasaurus
Moabosaurus
Lourinhasaurus
Haplocanthosaurus_delfsi
Haplocanthosaurus_priscus
Chuanjiesaurus
Cetiosauriscus
Bellusaurus
Soriatitan
Puertasaurus
Argentinosaurus
Patagotitan
Zby
Turiasaurus
Losillasaurus
Padillasaurus
Jobaria
Tapuiasaurus
Nemegtosaurus
Xianshanosaurus
Venenosaurus
Tendaguria
Tehuelchesaurus
Tastavinsaurus
Sonorasaurus
Sauroposeidon
Haestasaurus
Mongolosaurus
Malarguesaurus
Lusotitan
Liubangosaurus
Lapparentosaurus
Janenschia
Huanghetitan_ruyangensis
Huanghetitan
Wamweracaudia
Gobititan
Giraffatitan
Galveosaurus
Fusuisaurus
Fukuititan
Vouivria
Europasaurus
Euhelopus
Dongbeititan
Daxiatitan
Cloverly_titanosauriform
Cedarosaurus
Brachiosaurus
Baotianmansaurus
Australodocus
Atlasaurus
Astrophocaudia
Aragosaurus
Angolatitan
Abydosaurus
Diplodocus
Apatosaurus
Nigersaurus
Camarasaurus
Mamenchisaurus
Omeisaurus
Tangvayosaurus
Chubutisaurus
Ligabuesaurus
Phuwiangosaurus
Qiaowanlong
Paluxysaurus
Savannasaurus
Brontomerus
Epachthosaurus
Andesaurus
Ruyangosaurus
Dongyangosaurus
Rinconsaurus
Muyelensaurus
Notocolossus
Mendozasaurus
Jiangshanosaurus
Malawisaurus
Huabeisaurus
Rapetosaurus
Jainosaurus
Aeolosaurus
Isisaurus
Futalognkosaurus
Alamosaurus
Saltasaurus
Opisthocoelicaudia
Australotitan
Erketu
Pitekunsaurus
Wintonotitan
AODF_836
Diamantinasaurus
J
Royo-Torres et al. (2021) Dataset
Appendicular Only
99,999 Max Trees
TBR 1000 reps / 100 trees per rep
Strict Consensus Tree
Implied Weights K = 9
100 MPTs
54.54 steps
Shunosaurus
Turiasaurus
Losillasaurus_San_Lorenzo_
Losillasaurus_giganteus
Losillasaurus_Holotype
Narindasaurus
Amanzia
Amphicoelias
Supersaurus
Dinheirosaurus
Tornieria
Leinkupal
Zapalasaurus
Tataouinea
Rebbachisaurus
IOW_rebbachisaurid_caudal
Demandasaurus
Rayososaurus
Nopcsaspondylus
Katepensaurus
El_Chocon_rebbachisaurid
Cathartesaura
Limaysaurus
Histriasaurus
Comahuesaurus
Amazonsaurus
Suuwassea
Lingwulong
Brachytrachelopan
Amargasaurus
Dicraeosaurus
Mierasaurus
Moabosaurus
Lourinhasaurus
Haplocanthosaurus_delfsi
Haplocanthosaurus_priscus
Chuanjiesaurus
Cetiosauriscus
Bellusaurus
Soriatitan
Puertasaurus
Argentinosaurus
Patagotitan
Pitekunsaurus
Notocolossus
Mendozasaurus
Zby
Padillasaurus
Jobaria
Wintonotitan
Tapuiasaurus
Xianshanosaurus
Venenosaurus
Tendaguria
Tehuelchesaurus
Tastavinsaurus
Sonorasaurus
Sauroposeidon
Ruyangosaurus
Qiaowanlong
Phuwiangosaurus
Haestasaurus
Mongolosaurus
Malarguesaurus
Lusotitan
Liubangosaurus
Lapparentosaurus
Janenschia
Huanghetitan_ruyangensis
Huanghetitan
Wamweracaudia
Gobititan
Giraffatitan
Galveosaurus
Fusuisaurus
Vouivria
Europasaurus
Euhelopus
Erketu
Dongyangosaurus
Dongbeititan
Daxiatitan
Cedarosaurus
Brachiosaurus
Baotianmansaurus
Australodocus
Atlasaurus
Astrophocaudia
Aragosaurus
Abydosaurus
Diplodocus
Apatosaurus
Nigersaurus
Camarasaurus
Mamenchisaurus
Omeisaurus
Rinconsaurus
Muyelensaurus
Savannasaurus
Brontomerus
Epachthosaurus
Andesaurus
Paluxysaurus
Cloverly_titanosauriform
Australotitan
AODF_836
Diamantinasaurus
Ligabuesaurus
Fukuititan
Angolatitan
Tangvayosaurus
Chubutisaurus
Malawisaurus
Aeolosaurus
Rapetosaurus
Isisaurus
Futalognkosaurus
Nemegtosaurus
Opisthocoelicaudia
Saltasaurus
Jiangshanosaurus
Alamosaurus
Australia
South America
Asia

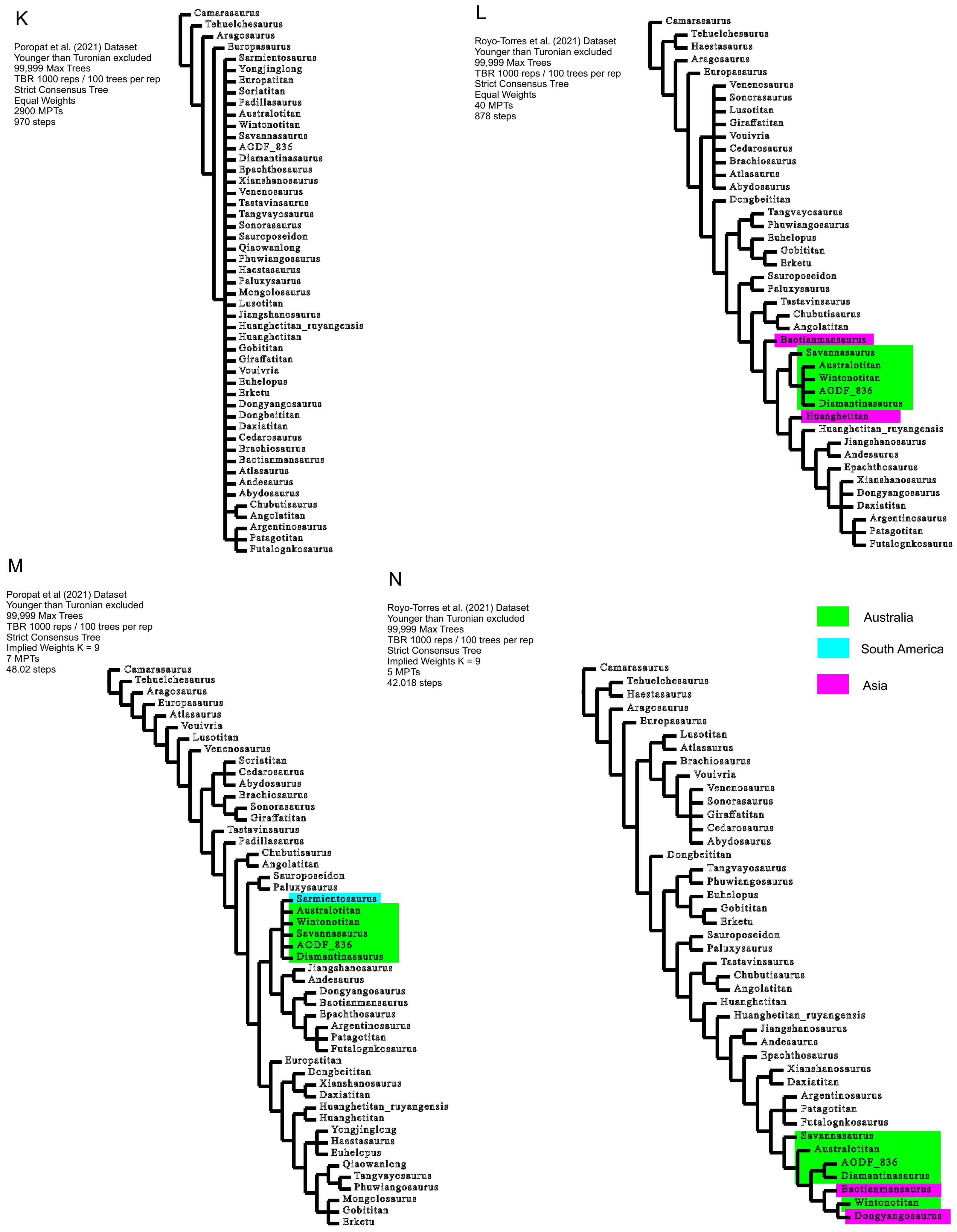
K
Poropat et al. (2021) Dataset
Younger than Turonian excluded
99,999 Max Trees
TBR 1000 reps / 100 trees per rep
Strict Consensus Tree
Equal Weights
2900 MPTs
970 steps
Camarasaurus
Tehuelchesaurus
Aragosaurus
Europasaurus
Sarmientosaurus
Yongjinglong
Europatitan
Soriatitan
Padillasaurus
Australotitan
Wintonotitan
Savannasaurus
AODF_836
Diamantinasaurus
Epachthosaurus
Xianshanosaurus
Venenosaurus
Tastavinsaurus
Tangvayosaurus
Sonorasaurus
Sauroposeidon
Qiaowanlong
Phuwiangosaurus
Haestasaurus
Paluxysaurus
Mongolosaurus
Lusotitan
Jiangshanosaurus
Huanghetitan_ruyangensis
Huanghetitan
Gobititan
Giraffatitan
Vouivria
Euhelopus
Erketu
Dongyangosaurus
Dongbeititan
Daxiatitan
Cedarosaurus
Brachiosaurus
Baotianmansaurus
Atlasaurus
Andesaurus
Abydosaurus
Chubutisaurus
Angolatitan
Argentinosaurus
Patagotitan
Futalognkosaurus
L
Royo-Torres et al. (2021) Dataset
Younger than Turonian excluded
99,999 Max Trees
TBR 1000 reps / 100 trees per rep
Strict Consensus Tree
Equal Weights
40 MPTs
878 steps
Camarasaurus
Tehuelchesaurus
Haestasaurus
Aragosaurus
Europasaurus
Venenosaurus
Sonorasaurus
Lusotitan
Giraffatitan
Vouivria
Cedarosaurus
Brachiosaurus
Atlasaurus
Abydosaurus
Dongbeititan
Tangvayosaurus
Phuwiangosaurus
Euhelopus
Gobititan
Erketu
Sauroposeidon
Paluxysaurus
Tastavinsaurus
Chubutisaurus
Angolatitan
Baotianmansaurus
Savannasaurus
Australotitan
Wintonotitan
AODF_836
Diamantinasaurus
Huanghetitan
Huanghetitan_ruyangensis
Jiangshanosaurus
Andesaurus
Epachthosaurus
Xianshanosaurus
Dongyangosaurus
Daxiatitan
Argentinosaurus
Patagotitan
Futalognkosaurus
M
Poropat et al (2021) Dataset
Younger than Turonian excluded
99,999 Max Trees
TBR 1000 reps / 100 trees per rep
Strict Consensus Tree
Implied Weights K = 9
7 MPTs
48.02 steps
Camarasaurus
Tehuelchesaurus
Aragosaurus
Europasaurus
Atlasaurus
Vouivria
Lusotitan
Venenosaurus
Soriatitan
Cedarosaurus
Abydosaurus
Brachiosaurus
Sonorasaurus
Giraffatitan
Tastavinsaurus
Padillasaurus
Chubutisaurus
Angolatitan
Sauroposeidon
Paluxysaurus
Sarmientosaurus
Australotitan
Wintonotitan
Savannasaurus
AODF_836
Diamantinasaurus
Jiangshanosaurus
Andesaurus
Dongyangosaurus
Baotianmansaurus
Epachthosaurus
Argentinosaurus
Patagotitan
Futalognkosaurus
Europatitan
Dongbeititan
Xianshanosaurus
Daxiatitan
Huanghetitan_ruyangensis
Huanghetitan
Yongjinglong
Haestasaurus
Euhelopus
Qiaowanlong
Tangvayosaurus
Phuwiangosaurus
Mongolosaurus
Gobititan
Erketu
N
Royo-Torres et al. (2021) Dataset
Younger than Turonian excluded
99,999 Max Trees
TBR 1000 reps / 100 trees per rep
Strict Consensus Tree
Implied Weights K = 9
5 MPTs
42.018 steps
Australia
South America
Asia
Camarasaurus
Tehuelchesaurus
Haestasaurus
Aragosaurus
Europasaurus
Lusotitan
Atlasaurus
Brachiosaurus
Vouivria
Venenosaurus
Sonorasaurus
Giraffatitan
Cedarosaurus
Abydosaurus
Dongbeititan
Tangvayosaurus
Phuwiangosaurus
Euhelopus
Gobititan
Erketu
Sauroposeidon
Paluxysaurus
Tastavinsaurus
Chubutisaurus
Angolatitan
Huanghetitan
Huanghetitan_ruyangensis
Jiangshanosaurus
Andesaurus
Epachthosaurus
Xianshanosaurus
Daxiatitan
Argentinosaurus
Patagotitan
Futalognkosaurus
Savannasaurus
Australotitan
AODF_836
Diamantinasaurus
Baotianmansaurus
Wintonotitan
Dongyangosaurus
